# Progress in Orthopædic Surgery

**Published:** 1904-04-30

**Authors:** 


### PROGRESS IN ORTHOPÆDIC SURGERY.

**Congenital Dislocation of the Hip.**—In a lecture given by A. H. Tubby,[1] he says that it is necessary to define the term "congenital dislocation," many of the cases being undoubtedly due to trauma during parturition. These are acquired, and not congenital dislocations, the joint being perfectly developed, and violent displacement have occurred. Congenital displacements are not very common in comparison with other joint affections. The condition is seen seven times as frequently in females as in males, and may be unilateral or bilateral. Congenital displacement of the head of the femur dates from intra-uterine life, and may be observed immediately after birth, no sign of traumatism being necessarily present. The deformity is hereditary, though examples of this are rare. It is invariably associated with changes in the acetabulum, but not, in the developmental state, with alterations in the head of the femur. The changes in the acetabulum are as follows:—In depth it is frequently shallow, scarcely large enough to admit the tip of the index finger; in shape nearly circular, or, more often, triangular. Sometimes it is entirely absent; sometimes its place is occupied by a convex mass of bone. Frequently the postero-superior border is deficient. The subsequent changes in the part are entirely due to want of adaptation. At birth the head of the femur is generally normal in shape, but probably from the time of birth, and certainly from the time the child begins to walk, it undergoes the following changes: It loses its circular outline, and becomes flattened; it is oval from above downwards, and sometimes irregular. In adult life it is frequently mushroom-like, and in old age it may disappear entirely. In some cases the growth of the head is retarded to a marked degree; in others the neck becomes shortened and altered in direction. As age advances the angle between the neck and the shaft of the femur becomes less obtuse. Further, the neck is atrophied, and appears to be an isthmus connecting the head with the great trochanter. It also undergoes version in such a way as to become curved anteriorly or posteriorly. It is therefore evident that treatment should be begun before the child has walked, and that if it is not commenced till late, there is less prospect of a satisfactory result, on account of the changes in the bony parts. The round ligament has been found absent in about 83 per cent. of the cases, in others it has been thick and solid, or elongated and attenuated. The capsular ligament is usually elongated; sometimes thickened, and may have served to support the whole weight of the body. In some cases the cavity of the joint is dilated, in others has a curious hour-glass shape, and is obliterated in the middle. The muscles passing from the pelvis to the great trochanter are lengthened, while the adductors are shortened. The ilio-psoas, instead of lying over the head and neck of the femur, comes to pass more and more internally, and is situated over the anterior border of the cotyloid cavity, and finally passes over the posterior border. The direction of the displacement is usually upwards and backwards, but a considerable number of anterior displacements are met with. The formation of an osteo-fibrous joint is possible, but the chief obstacles to the formation of a new joint capable of supporting the body weight are (1) the upward thrust, tending to push the head of the femur further out of place; (2) the interposition, between the periosteum of the ilium and the head of the femur of the thickened capsule and of the elongated round ligament, and (3) the constant adduction of the femur, so that its head loses its full contact with the iliac bone. As regards the symptoms: the patient walks with a waddling gait, but this is less marked when he is running. Much lordosis is present, and when the patient stands the hands reach much lower down on the thighs than in an ordinary individual. The muscles of the extremity are small and ill-developed, and there is a want of proportion between the size of the body and lower limbs, the pelvis itself appearing broader than usual. On standing, the great trochanters are angular and unduly prominent, and displaced upwards. The gluteal muscles form a cone with the apex at the great trochanter, the heels are rotated inwards, and the toes turned outwards. On placing the patient on the back the lordosis disappears, and the distance between the tops of the trochanters and the crests of the ilia becomes greater. The negative signs are—total absence of pain, no limitation of movement in young children, and little limitation in adolescence. In making the diagnosis it is necessary to be certain that the displacement is really congenital and not due to severe traumatism at birth. If the affection is bilateral, it is easier to form an opinion than when it is unilateral. Confusion has arisen between congenital dislocation and coxa vara, but the best test is the use of the *x*-rays in a doubtful case. The diagnosis has also sometimes to be made from pseudo-hypertrophic paralysis, and frequently from morbus coxæ, and in cases where the child has not walked there is often difficulty in distinguishing congenital from paralytic dislocation, and there can be no doubt that many so-called congenital displacements are in reality paralytic. The object of treatment should be the complete removal of the deformity, the substitution of a sound joint for the loose and telescopic one, and the removal of the waddling gait. Three methods deserve notice: (1) prolonged recumbency with reduction; (2) reduction by the open method, as formerly practised by Hoffa and Lorenz; (3) forcible extension and reduction by manipulation, according to the method originally advocated by Paci, modified by Hoffa and Lorenz. But no mode of treatment gives perfect results. The first method has many disadvantages, such as the long duration of recumbency and of the after treatment, and interference with the general health. At least four years' constant supervision is required, and much expense, the result frequently being disappointing. Treatment by the method of open operation was first advocated by Hoffa, but his method has now been discarded on account of its extreme severity and danger. Lorenz, instead of cutting so widely as Hoffa, made only an anterior incision between the sartorius and the tensor vaginæ femoris, but few of his cases appear to have been entirely successful. It may be noted that Lorenz has abandoned this method, and now uses forcible reduction whenever possible. Forcible reduction by manipulation was first practised by Paci, and strongly advocated and practised by Mr. Reeves. It is performed in the following manner: The patient is placed recumbent, and the surgeon flexes the leg fully on the thigh, and the thigh on the pelvis, thus carrying the head of the femur downwards. The thigh is abducted fully, the limb strongly rotated outwards, and then extended fully. If the displacement be anterior, the limb should be abducted and rotated inwards. The limb is put up in plaster of Paris bandages for a month, followed by continuous extension for three months. Then walking may be commenced on crutches. Lorenz adopted this method with some modifications, but the procedure which usually is associated with his name, is not above criticism, as very disastrous results have sometimes followed, owing to the violence employed, such as gangrene, fractures, and persistent paralysis of the sciatic nerve. Mr. Tubby considers that, on the whole, Paci's method of operation, as modified by Lorenz, promises the best results, but that these are not as good as they ought to be, and further earnest work is greatly needed on this subject. In a paper presented at a meeting of the American Orthopædic Society, Dr. E. H. Bradford,[2] of Boston, said that he was convinced that Lorenz's method of treatment of these cases (between the ages of three and six) was a satisfactory one. He was of opinion that less resistance was offered by the capsule than by the muscles and their tendons, the chief resistance being from the strong tendon of the adductor longus. In very young children manual manipulation by Lorenz's method was sufficient; but in the resistent cases mechanical force, applied so as to pull upon and abduct the limb, while acting directly upon the capsule, was of assistance. Where an immoderate amount of force was required, owing to the strong resistance of the adductors, he considered it advisable to divide the chief resisting structures, rather than incur the danger of fracturing the femur or severely bruising the tissues.

**Radiographs Illustrating Congenital Dislocation of the Hip.**—Dr. Morgan[3] considers that radiography should be of assistance to the surgeon in the selection of suitable cases for reduction by manipulation, and in obtaining an accurate knowledge of the position of the head during the after-treatment of these cases. For the proper interpretation of any radiograph the surgeon must know the position of the limbs during exposure, and the relative position of plate and tube. A large proportion of radiographs show either absence of the neck of the femur or a very twisted short one, these appearances being wholly due to the position in which the limb is held as regards in or out rotation. Mr. Robert Jones permitted Dr. Morgan to radiograph several of his patients before, during, and after reduction by the method of Lorenz, so as to show the different appearances exhibited in the femoral head and neck, dependent upon rotation of the limbs, and figures are given showing some of the results.

**Peripheral Palsies following Manual Replacement of the Congenitally Dislocated Hip.**—Dr. Henry Ling Taylor,[4] of New York has observed that in many of the cases operated upon by the method of Lorenz, the children were not always able to get up and walk within a few days or weeks as was desirable, and examination of these cases showed complete loss of power in the quadriceps muscle, and therefore of extension at the knee. On further inquiry he found that nine cases of peripheral palsy of the quadriceps had followed the operation. These patients had since been examined at intervals, and had either completely recovered from the palsy, or were recovering without treatment.

[1] Clin. Jour., July 1, 1903, p. 168. [2] Med. Rec., Aug. 15, 1903, p. 277. [3] Brit. Med Jour., April 11, 1903, p. 842. [4] Med. Rec., Aug. 15, 1903, p. 277.

*(To be continued.)*

